# The Common Traits of the ACC and PFC in Anxiety Disorders in the DSM-5: Meta-Analysis of Voxel-Based Morphometry Studies

**DOI:** 10.1371/journal.pone.0093432

**Published:** 2014-03-27

**Authors:** Jing Shang, Yuchuan Fu, Zhengjia Ren, Tao Zhang, Mingying Du, Qiyong Gong, Su Lui, Wei Zhang

**Affiliations:** 1 Mental Health Center, Department of Psychiatry, West China Hospital of Sichuan University, Chengdu, People's Republic of China; 2 Radiology Department of the Second Affiliated Hospital, Wenzhou Medical University, Wenzhou, People's Republic of China; 3 West China School of Public Health, Sichuan University, Chengdu, People's Republic of China; 4 Huaxi MR Research Center (HMRRC), Department of Radiology, West China Hospital of Sichuan University, Chengdu, People's Republic of China; University of Electronic Science and Technology of China, China

## Abstract

**Background:**

The core domains of social anxiety disorder (SAD), generalized anxiety disorder (GAD), panic disorder (PD) with and without agoraphobia (GA), and specific phobia (SP) are cognitive and physical symptoms that are related to the experience of fear and anxiety. It remains unclear whether these highly comorbid conditions that constitute the anxiety disorder subgroups of the Diagnostic and Statistical Manual for Mental Disorders – Fifth Edition (DSM-5) represent distinct disorders or alternative presentations of a single underlying pathology.

**Methods:**

A systematic search of voxel-based morphometry (VBM) studies of SAD, GAD, PD, GA, and SP was performed with an effect-size signed differential mapping (ES-SDM) meta-analysis to estimate the clusters of significant gray matter differences between patients and controls.

**Results:**

Twenty-four studies were eligible for inclusion in the meta-analysis. Reductions in the right anterior cingulate gyrus and the left inferior frontal gyrus gray matter volumes (GMVs) were noted in patients with anxiety disorders when potential confounders, such as comorbid major depressive disorder (MDD), age, and antidepressant use were controlled for. We also demonstrated increased GMVs in the right dorsolateral prefrontal cortex (DLPFC) in comorbid depression-anxiety (CDA), drug-naïve and adult patients. Furthermore, we identified a reduced left middle temporal gyrus and right precentral gyrus in anxiety patients without comorbid MDD.

**Conclusion:**

Our findings indicate that a reduced volume of the right ventral anterior cingulate gyrus and left inferior frontal gyrus is common in anxiety disorders and is independent of comorbid depression, medication use, and age. This generic effect supports the notion that the four types of anxiety disorders have a clear degree of overlap that may reflect shared etiological mechanisms. The results are consistent with neuroanatomical DLPFC models of physiological responses, such as worry and fear, and the importance of the ventral anterior cingulate (ACC)/medial prefrontal cortex (mPFC) in mediating anxiety symptoms.

## Introduction

Social anxiety disorder (SAD), generalized anxiety disorder (GAD), panic disorder (PD), agoraphobia (AG), and specific disorder(SP) are major anxiety disorders identified by the Anxiety, OC Spectrum, Posttraumatic, and Dissociative Disorders working group [1]. According to the National Comorbidity Survey, the overall lifetime prevalence of anxiety disorders is 24.9%, which includes rates of 13.3%, 5.1%, and 3.5% for SAD, GAD, and PD with and without AG, respectively [2]. Although they present with distinct features, the disorders are also comorbid and share common clinical features, such as extensive anxiety, physiological anxiety symptoms, behavioral disturbances, and associated distress or impairment.

It is still unknown whether these conditions represent distinct disorders or alternative presentations of a single underlying pathology. Structural magnetic resonance imaging (MRI) studies are potentially more amenable to comparisons across anxiety disorders because they are paradigm-free. Their merits allow voxel-based morphometry (VBM) studies to contribute to a better understanding of the neurobiology of psychiatric disorders. Unfortunately, recent applications of this novel method have often been limited by relatively small sample sizes, which result in insufficient statistical power and inconsistent conclusions. There are few studies on structural abnormalities in specific anxiety disorders, namely, SAD, GAD, and SP [3]. In a recent meta-analysis of data by Chien-Han Lai, quantitative morphometric MRI studies were analyzed and revealed decreased regional gray matter volumes in the right caudate head and right parahippocampal gyrus in the brains of PD patients [4]. However, the results were potentially biased by the inclusion of only 6 studies. Another meta-analyses by Radua et al [5] found other anxiety disorders and obsessive- compulsive disorder (OCD) has common as well as distinct neural substrates. The pathophysiology and structure deficits of these disorders require further study to support the pathogenic model. Importantly, only a few studies have explicitly controlled for depression comorbidity, although morphometric changes have also been identified in anxiety disorders [6].

The principal aim of this study was to perform a search of all the published VBM studies on SAD, GAD, PD, AG, and SP to better understand whether these frequently co-occurring anxiety disorders may have, at least in part, a common etiology by controlling for the effects of antidepressants and age as potential confounders. The current study applies a new version of the voxel-based meta-analytical technique, called effect-size signed differential mapping (ES-SDM), which uses well-established statistics to combine statistical parametric maps and peak coordinates. The method enables us to qualitatively compare gray matter differences to examine the extent to which these anxiety disorders share neural substrates. Recent data suggest that depression and anxiety have a similar etiology and respond to the same treatment strategies [7]. There have also been claims that a similar neural, and presumably computational, architecture mediates symptoms of mood and anxiety [8]. However, based on previous suggestions that comorbid depression may affect the anatomical features of anxiety disorders, it is necessary to conduct an analysis to assess the results without comorbid depression. The pathophysiology and structural deficits associated with anxiety disorders with and without comorbid depression require further study to support the pathogenic model. The second aim of this study was to investigate the shared and unique neuroanatomical profile of anxiety and comorbid depression by discarding the patients who currently exhibit depression. Based on previous studies, we hypothesized that anxiety patients with or without comorbid depression would exhibit changes in their gray matter volumes (GMVs) in the anterior cingulate (ACC) and prefrontal cortex (PFC), including the ventral and dorsal ACC and the inferior frontal gyrus (IFG), because those regions play a critical role in emotional processing, especially that of anxiety and fear [9]. In addition, we predicted that differences in the above regional GMVs would be found between anxiety disorder patients and healthy controls (HCs) after excluding children and adolescents (aged<18 years) and the patients who were taking medication.

## Methodology

### Enrollment of Studies

Potentially eligible studies that examined individuals with SAD, GAD, PD, AG, and SP were identified by conducting a search of the PubMed, ScienceDirect, and EBSCO databases between January 2001 (the date of the first VBM study in any anxiety disorder) and November 2013 using a combination of the following terms: “social anxiety” or “social phobia” or “SAD”, “generalized anxiety disorder” or “GAD”, “panic disorder” or “PD”, “specific phobia” or “SP”, “agoraphobia”, “phobia”, “stress disorder”, “anxiety”, and “Voxel-Based Morphometry” or “VBM” or “voxelwise”. The study inclusion was restricted to whole-brain studies that provided standard Talairach or Montreal Neurologic Institute (MNI) coordinates, which are necessary for a voxel-level quantitative meta-analysis. The SAD, PD, and GA studies that were included focused on adult subjects, and the GAD studies focused on children and adolescent participants. Because GA is not an independent disease category in the Diagnostic and Statistical Manual for Mental Disorders, fourth edition (DSM-IV) [10], we combined GA and PD in this study. To our knowledge, no VBM research on specific phobias has been reported. Owing to the small overall number of VBM studies, we included studies with participants from all age groups in the meta-analysis.

### Selection Process

The selection process consisted of three stages. Two independent reviewers first assessed the titles of the search results and retrieved the relevant articles. Second, the articles that remained eligible were assessed based on their abstract to determine whether any of the inclusion criteria were not met. The full text of all remaining articles was then assessed with a data extraction template, which was constructed for the purpose of organizing and extracting information from the included articles and excluding articles without peak values. After examining the title and abstract, a total of 24 studies fulfilled all the inclusion criteria.

### Data Synthesis and Effect-Size Signed Differential Mapping

The regional differences in the GMV between the patients and controls were analyzed using an effect-size signed differential mapping (ES-SDM; http://www.sdmproject) [11]. First, we ensured that the same threshold was used throughout the entire brain in each included study to avoid biases toward liberally thresholded brain regions. Second, an effect-size signed map of the differences in gray matter was separately recreated for each study. In contrast to other methods, such as activation likelihood estimation (ALE) and multilevel kernel density analysis (MKDA), ES-SDM uses both the reported peak coordinates and the effect sizes, which enables the reported peak coordinates to be combined with statistical parametric maps to allow for a more detailed and accurate meta-analysis [11]. Third, the mean map was obtained by calculating the mean of the study maps, weighting the means by the inverse of each study variance and accounting for inter-study heterogeneity. The statistical significance was assessed by performing a permutation test.

### Data Extraction

The data that were extracted included (a) author names, (b) date of publication, (c) disease categories and brain atlas, (d) subject group numbers, (e) comorbidities, (f) medications used (including past and current antidepressants), (g) mean age with standard deviation, (h) gender ratio, and (i) coordinates associated with larger or smaller GMVs in the anxiety disorder patients compared with the HCs. A Gaussian kernel with a 20-mm half-width was employed to assign indicators of proximity to the reported coordinates because it can optimally balance sensitivity and specificity in ES-SDM [11]. We used the standard ES-SDM thresholds (uncorrected P<0.005, threshold extent of clusters>10 voxels), which were proposed to optimally balance sensitivity and specificity and to be approximately equivalent to the corrected P value for effect size of 0.05 in ES-SDM [11].

Finally, to investigate the potential confounding effect of comorbid depression, age, and antidepressant treatment, we performed meta-analytical comparisons between sub-groups of coordinates (separately discarding the patients who exhibited comorbid depression, currently used medication, and were<18 years old). These comparisons demonstrated that there were no significant differences in the GMVs of various regions in each group. The SDM value and number of voxels in the case vs. control comparison performed in this meta-analysis are reported.

We illustrate the results using MRICron (http: //www.mccauslandcenter. sc.edu/mricro/mricron/) with Talairach coordinates.

## Results

### Anxiety patients with comorbid depression

**Figure 1:**
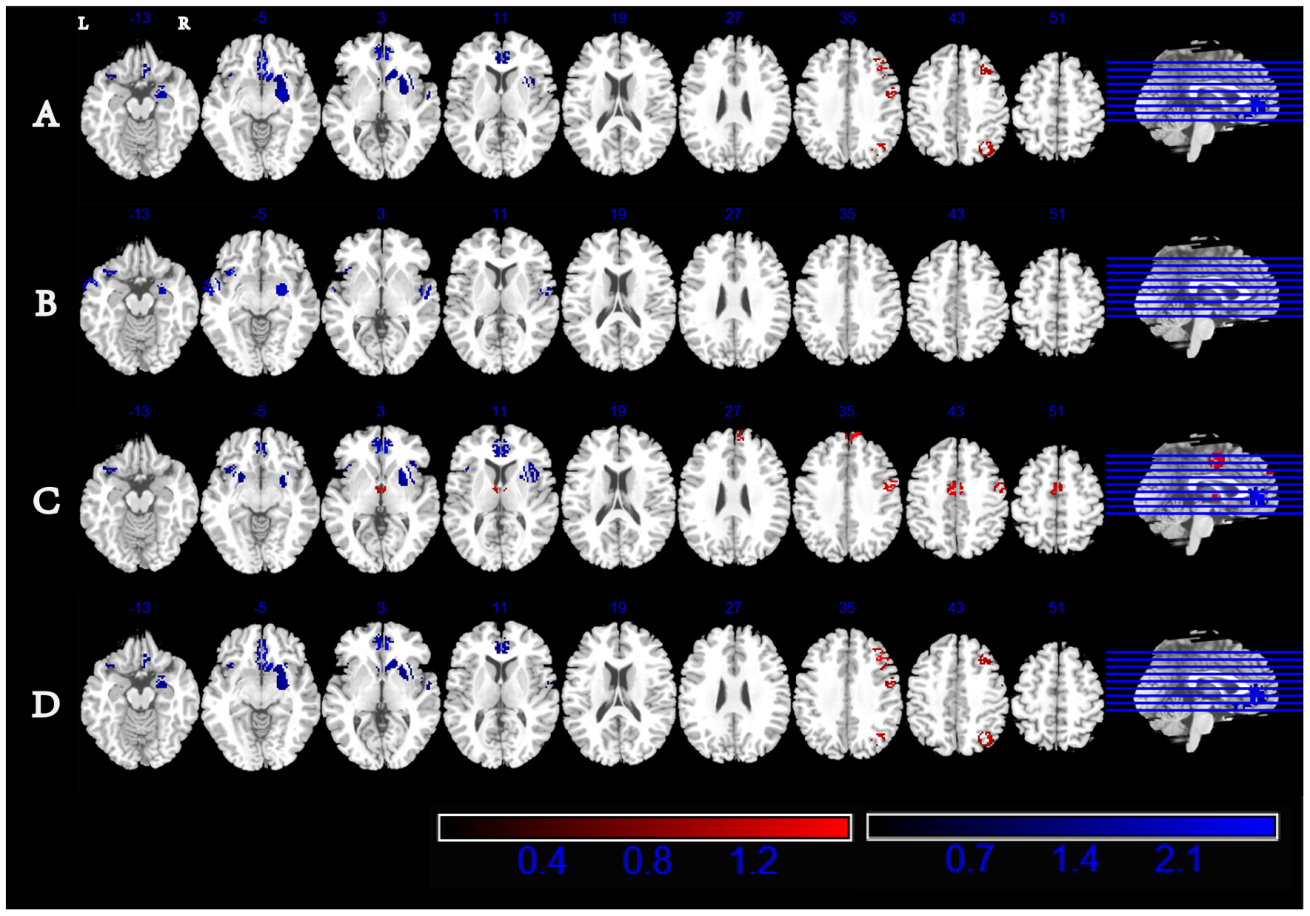
SDM map investigating changes of GMV in anxiety patients comorbid depression (A), anxiety patients without comorbid depression(B), drug-naïve patients(C) and adult patients(D) with axial sections. All coordinates are in the Talairach space and clusters are displayed using the standard ES-SDM thresholds (uncorrected P<0.005, extent threshold of clusters>10 voxels). Areas with increased gray matter relative to controls are displayed in red, and areas with decreased gray matter are displayed in blue. L, left; R, right.

Twenty-four studies were included in this meta-analysis according to the search criteria mentioned above and consisted of 5 studies on SAD [12]–[16]; 3 studies on GAD [17]–[19]; 13 studies on PD with and without GA [12], [20]–[31]; 2 studies on MDD and PD [32], [33]; 1 study on SAD, GAD, and separation anxiety disorder [34]; and 1 study on SAD, GAD, and PD with and without MDD [6]. These studies yielded a total combined sample of 617 subjects with comorbid depression-anxiety (CDA) and 647 HCs. The detailed information on the study characteristics and subject demographics is summarized in *Table 1*. The results from the ES-SDM analysis are summarized in *Table 2*. The axial sections of the ES-SDM volumetric reconstructions of the anxiety patients showed decreased volumes in the right ventral anterior cingulate and left inferior frontal gyrus and increased volumes in the right precentral gyrus, right middle frontal gyrus, and right inferior parietal lobule compared with those of the HCs (*Table 2* *,* *Figure 1*).

**Table 1:** Characteristics of included studies.

Author	Disease category/Brain atlas	Cases (F/M)	Comorbility	Medication used	Age (Years)	Controls (F/M)	Age (Years)
1.Ardesheer Talati et al (2013)^10^	SAD/MNI SPM	33(24/9)	MDD11,GAD5,SP4,OCD1, DUD1,AUD2,Lifetime Psych. Medication Use9	N/A	31.5(8.2)	37(19/18)	31.4 (9.1)
1.*Ardesheer Talati et al (2013)^10^	PD/MNI SPM	16(13/3)	MDD3, GAD2, SP5, OCD2, DUD4, AUD4, Lifetime Psych. Medication Use9	N/A	31.8 (10.0)	20(9/11)	31.4 (7.8)
2.Wei Liao et al (2011)^11^	SAD/MNI SPM	18(6/12)	None	None	22.7(3.8)	18(5/13)	21.9(3.7)
3.Yajing Meng et al (2013)^12^	SAD/MNI SPM	20(6/14)	None	None	21.8(3.7)	19(6/13)	21.6(3.77)
4.Andreas Frick et al (2013)^13^	SAD/MNI SPM	14(0/14)	OCD2, SP1	Venlafaxine1, varenicline tartrate1	32.6(8.7)	12(0/12)	27.9(7.9)
5.Annette Beatrix et al (2013)^14^	SAD/MNI/FSL	46(17/29)	MDD1	Antidepressants19	33.0(8.9)	46(17/29)	33.1(10.6)
6.Mei Liao et al (2013)^15^	GAD/MNI SPM	26(13/13)	None	None	16–18	25(12/13)	16–18
7.Jeffrey R. Strawn et al (2013)^16^	GAD/MNI SPM	15(8/7)	ADHD6, SAD4,SP1	None	13(2)	28(17/11)	13(2)
8.Anne Schienle et al (2011)^17^	GAD/MNI SPM	16(16/0)	None	None	22.9(4.1)	15(15/0)	23.7(3.7)
9.Guillem Massana et al (2003)^18^	PD/TAL SPM	18(11/7)	AG15	None	36.8(11.3)	18(10/8)	36.7(8.8)
10. Hani k K. Yoo et al (2005)^19^	PD/TAL SPM	18(9/9)	None	previous exposures to antidepressants10	33.3(7.1)	18(7/11)	32.0(5.8)
11.Xenia Pro topopescu et al (2006)^20^	PD/MNI SPM	10(6/4)	AG2, past alcohol abuse1, past alcohol dependence1	None	35.5(9.7)	23(11/12)	28.7(7.5)
12.Ricardo R. Uchida et al (2008)^21^	PD/TAL SPM	19(16/3)	AG14, MDD3, dysthymia 2, past MDD 9	antidepressant15,BEN1,antidepressant 14 +BEN4	37.1(9.8)	20(16/4)	36.5(9.9)
12.*Ricardo R. Uchida et al (2008)^21^	PD/TAL SPM	14	without current MDD	N/A	N/A	20(16/4)	36.5(9.9)
13. Takeshi Asami et al (2008)^28^	PD/TAL SPM	26(16/10)	Past MDD 4, past AG11; dysthymia1, AG3 at the time of the study	antidepressants21(SSRI17, SNRI2, tricycles2),BEN19	37.7(10.1)	26(16/10)	38.2(9.7)
14.Takeshi Asami et al (2009)^22^	PD/TAL SPM	24(15/9)	Past MDD 3, past AG13; dysthymia1, AG3 at the time of the study	antidepressants19 (SSRI15, SNRI2, tricycles2),BEN17	37.0(10.0)	24(15/9)	37.0(8.7)
15.Fumi Hayano et al(2009)^23^	PD/MNI SPM	27(17/10)	past MDD3, AG11 at the time of the study	BEN4,SSRI3,SSRI+ SNRI1,BEN+SSRI12, BEN+SNRI2,BEN+TCA2,BEN+SSRI+ TCA1	38.2(9.9)	30(21/9)	35.3(10.5)
16.Tomohide Roppongi (2010)^24^	PD/MNI SPM	28(18/10)	AG15, MDD1, past dysthymia1, past MDD6	Antidepressants+ BEN 25	38.4(9.8)	28(18/10)	37.8(9.8)
16.*.Tomohide Roppongi (2010)^24^	absent-POS group	9	N/A	N/A	N/A	10	N/A
16.*.Tomohide Roppongi (2010)^24^	single-POS group	24	N/A	N/A	N/A	26	N/A
17.T. Sobanski et al (2010)^25^	PD/MNI SPM	17(9/8)	SAD1, GAD1, adjustment disorder with mixed disturbance of emotions and conduct2, PPD2, AG16	None	34.9(6.7)	17(9/8)	33.1(6.2)
18.Chien-Han Lai et al (2012)^26^	PD/MNI FSL	30(19/11)	None	None	47.0(10.6)	21(11/10)	41.1(11.8)
19.Kyoung-Sae Na et al (2012)^27^	PD/MNI SPM	22(9/13)	AG12	None	40.2(10.5)	22(11/11)	40.2(12.4)
19.* Kyoung-Sae Na et al (2012)^27^	AG/MNI SPM	12(7/5)	None	None	43.1(9.6)	22(11/11)	40.2(12.4)
20.Chien-Han Lai et al (2013)^29^	PD/MNI FSL	21(9/12)	None	escitalopram21,alpraz-olam for acute panic attacks	46.6(11.4)	21(10/11)	41.1(11.8)
21.Chien-Han Lai(2010)^30^	PD and MDD/MNI FSL	16(11/5)	AG6	None	37.9(8.7)	15(11/4)	34.3(9.9)
22.Chien-Han Lai et al (2011)^31^	PD and MDD/MNI FSL	15(10/5)	None	Duloxetine15	35.9(9.6)	15(11/4)	34.3(9.9)
23.Michael P. Milham et al(2005)^32^	/MNI SPM	17(9/8)	SAD7, separation anxiety disorder3, GAD13, MDD4	previous exposures to antidepressants2	12.9(2.3)	34(18/16)	12.4(2.2)
23.*Michael P. Milham et al(2005)^32^	/MNI SPM	13	SAD7, separation anxiety disorder3, GAD13	N/A	N/A	26	N/A
24.Marie-Jose'van Tol et al (2010)^33^	/TAL SPM	88(59/29)	MDD+GAD17,MDD+PD17,MDD+PD+GAD9,MDD+SAD9,MDD+SAD+GAD15,MDD+PD+SAD12,MDD+PD+SAD+GAD9	None	37.3(10.6)	65(41/24)	40.5 (9.7)
24.* Marie-Jose'van Tol et al (2010)^33^	/TAL SPM	68(50/18)	PD20,PD+GAD2;SAD25; SAD+GAD3;PD+SAD14; PD+ SAD+ GAD4	None	35.96 (9.45)	65(41/24)	40.54 (9.71)

TTM, trichotillomania; BDD, body dysmorphic disorder; AUD, alcohol use disorder (abuse or dependence); CON, control subjects; DUD, drug use disorder (abuse or dependence); GAD, generalized anxiety disorder; MDD, major depressive disorder; OCD, obsessive compulsive disorder; PD, panic disorder; SAD, social anxiety disorder; SP, specific phobia; AG, agoraphobia;SSRI, selective serotonin reuptake inhibitor; SNRI, serotonin and noradrenaline reuptake inhibitors; BEN, benzodiazepine; TCA, tetracyclic antidepressant.

*subgroup analysis within the same study as it showed with the same serial-number

**Table 2:** Regions With Significant Differences In Gray Matter Volumes Between Patients With Anxiety Disorders Comorbid Depression And Healthy Controls.

Regions	Maximum		Cluster		
	Talairach Coordinates	SDM value	P Value	No. of Voxels	Cluster breakdown
Clusters of increased gray matter					
Right precentral gyrusΔ	48, −4, 36	1.051	0.001284522	84	BA6
Right middle frontal gyrus	34, 24, 40	1.444	0.000038536	195	BA8
Right inferior parietal lobule	34, −62, 40	1.311	0.000102762	149	BA39
Clusters of decreased gray matter					
Right ventrl anterior cingulate*	4, 34, 4	−2.551	∼0	1462	BA24
Left inferior frontal gyrus*	−34, 16, −8	−1.340	0.002196532	68	BA47

*shared GM alterations in patients with anxiety disorders comorbid depression, without comorbid depression, drug-naïve patients and adult patients.

Δ shared increased GMV in patients with anxiety disorders comorbid depression, drug-naïve patients and adult patients, decreased GMV in anxiety patients without comorbid depression.

### Confounding factors

#### Anxiety Patients without Current Depression

Although anxiety and depression share symptoms that are typically represented by a negative affect (NA) or general distress factor [35], it was necessary to conduct an analysis to test the effects of anxiety by excluding 5 studies [12], [16], [26], [32], [33] that included patients with comorbid depression. Because 3 studies separately [6], [23], [34] reported the results of anxiety patients with and without depression, the above analyses were treated as two independent experiments and were calculated twice separately. The included studies constituted a total of 429 anxiety subjects and 470 HCs (from 3 SAD studies, 3 GAD studies, 11 PD with and without GA studies, 1 study that combined the above four types of anxiety disorders, and 1 study that included patients with SAD, GAD, and separation anxiety disorder). The results of the analyses not only showed decreased regional GMVs in the right ventral anterior cingulate and left inferior frontal gyrus, which are areas that are also associated with CDA, but also revealed decreased GMVs in the left middle temporal gyrus and right precentral gyrus (*Table 3* *,* *Figure 1*).

**Table 3:** Regions With Significant Differences In Gray Matter Volumes Between Patients With Anxiety Disorders Without Comorbid Depression And Healthy Controls.

Regions	Maximum		Cluster		
	Talairach Coordinates	SDM value	P Value	No. of Voxels	Cluster breakdown
Clusters of increased gray matter					
Clusters of decreased gray matter					
Right ventral anterior cingulate*	6, 20, −4	−2.175	0.000025690	687	BA24
Left inferior frontal gyrus*	−56, 2, −10	−1.903	0.000077071	224	BA47
Right precentral gyrusΔ	48, −8, 8	−1.457	0.000937701	90	BA6
Left middle temporal gyrus	−56, 2, −10	−1.903	0.000077071	224	BA21

*shared GM alterations in patients with anxiety disorders comorbid depression, without comorbid depression, drug-naïve patients and adult patients.

Δ shared increased GMV in patients with anxiety disorders comorbid depression, drug-naïve patients and adult patients, decreased GMV in anxiety patients without comorbid depression.

#### Drug-naïve anxiety patients

Recent studies have reported that antidepressant exposure seems to prevent atrophy of the frontal cortex in depressed subjects [22], [36]. To eliminate potential interference with our results, we excluded the 9 studies that included participants who were taking antidepressant medication [15], [16], [23]–[26], [30], [31], [33]. The results remained largely unchanged, with decreased GMVs found in the right ventral anterior cingulate and left inferior frontal gyrus, which are regions that were also found to exhibit decreased GMVs in CDA. Furthermore, the results also revealed a decreased GMV in the right insula and increased GMVs in the right precentral gyrus, right thalamus, right medial frontal gyrus, and left dorsal cingulate gyrus compared with healthy subjects (*Table 4* *,* *Figure 1*).

**Table 4:** Regions With Significant Differences In Gray Matter Volumes Between Drug-naïve Patients With Anxiety Disorders Comorbid Depression And Healthy Controls.

Regions	Maximum		Cluster		
	Talairach Coordinates	SDM value	P Value	No. of Voxels	Cluster breakdown
Clusters of increased gray matter					
Right thalamus	2, −6, 4	1.539	∼0	229	Right thalamus
Right precentral gyrusΔ	44, −2, 36	1.163	0.000488118	175	BA6
Left dorsal cingulate gyrus	−2, −6, 40	1.323	0.000154143	299	BA24
Right medial frontal gyrus	6, 52, 36	1.230	0.000256904	130	BA9
Clusters of decreased gray matter					
Right ventrl anterior cingulate*	2, 38, 6	−2.604	∼0	438	BA24
Left inferior frontal gyrus*	−30, 14, −12	−1.478	0.001592807	216	BA47
Right insula	32, 14, 10	−1.951	0.000115607	417	BA13

*shared GM alterations in patients with anxiety disorders comorbid depression, without comorbid depression, drug-naïve patients and adult patients.

Δ shared increased GMV in patients with anxiety disorders comorbid depression, drug-naïve patients and adult patients, decreased GMV in anxiety patients without comorbid depression.

#### Adult Patients with Anxiety Disorders

After the exclusion of 3 studies [17], [18], [34] including childhood and adolescent subjects (age<18 years), the results of the adult anxiety subjects (age>18 years) were found to be similar to the outcomes of CDA patients, who exhibited decreased GMVs in the right ventral anterior cingulate and left inferior frontal gyrus and increased GMVs in the right precentral gyrus, right medial frontal gyrus, and right inferior parietal lobule. The age effect did not significantly influence the results of this study (*Table 5* *,* *Figure 1*).

**Table 5:** Regions With Significant Differences In Gray Matter Volumes Between Adult Patients With Anxiety Disorders Comorbid Depression And Healthy Controls.

Regions	Maximum		Cluster		
	Talairach Coordinates	SDM value	P Value	No. of Voxels	Cluster breakdown
Clusters of increased gray matter					
Right medial frontal gyrus	38, 24, 36	1.546	0.000038536	235	BA9
Right precentral gyrusΔ	48, −4, 36	1.119	0.001168915	84	BA6
Right inferior parietal lobule	34, −62, 42	1.330	0.000115607	156	BA39
Clusters of decreased gray matter					
Right ventral anterior cingulate*	4, 34, 4	−2.714	∼0	1344	BA24
Left inferior frontal gyrus*	−34, 16, −8	−1.378	0.002055235	74	BA47

*shared GM alterations in patients with anxiety disorders comorbid depression, without comorbid depression, drug-naïve patients and adult patients.

Δ shared increased GMV in patients with anxiety disorders comorbid depression, drug-naïve patients and adult patients, decreased GMV in anxiety patients without comorbid depression.

## Discussion

We present the first ES-SDM meta-analysis of VBM studies that examined the brain structural similarities of the anxiety subgroups of the DSM-5. After controlling for the effects of comorbid depression, antidepressants, and age, these anxiety disorders were found to share the same brain regions of decreased GMVs (namely, the right ventral ACC and left IFG) compared with controls. Therefore, the current pattern of results indicates that SAD, GAD, PD, and GA are not completely different entities at the level of their neuroanatomical phenotypes. This observation is simplistic, but lends further support to the argument that the anxiety subgroups share biological dimensions.

The shared decreased GMVs in the right ventral ACC and left inferior frontal gyrus in patients with mood and/or anxiety disorders suggest that the CDA group may have some GMV deficit overlaps with pure anxiety patients. We can hypothesize that these 2 areas possibly contribute to a final “atrophic” pathway leading to pure anxiety and CDA.

Convergent evidence for the functional differentiation between the ventral and dorsal ACC/mPFC is derived from “emotional conflict” research, in which the ACC is divided into ventral-rostral “regulation” and dorsal-caudal “appraisal/expression” subdivisions [9]. Amit Etkin et al [37] found that the ventral ACC is associated with the resolution of emotional conflict because of its interaction with the amygdala. The ventral ACC projects to the amygdala, and the amygdala in turn regulates various sites, including the sympathetic nervous system [38]. A previous meta-analysis showed that activation of the vACC/mPFC is associated with positive emotion, which can serve to regulate and diminish negative emotion [39]. The ventral ACC/mPFC may also perform a generic negative emotion inhibitory function whenever there is a need for the suppression of limbic reactivity [40]. In line with these results, we speculated that atrophy of the ventral ACC/mPFC is the main reason for the occurrence of anxiety disorders and that emotional processing cannot be modulated through deliberate and conscious application of top-down executive control over emotional stimulus processing and cannot inhibit fear that occurs during periods of anxiety.

Furthermore, our results showed increased GMVs in the right dorsolateral PFC (DLPFC, including Brodmann areas 6/8/9/46) [16] in CDA, drug-naïve, and adult patients. Previous neuroimaging studies upheld the long-held and popular view that both the DLPFC and dorsal ACC are implicated in emotion regulation circuits [16], [41], which supports the interpretation of a compensatory increase in the thickness of these regions in the current study. However, a recent review by Amit Etkin et al did not support this view and showed that the dorsal ACC/mPFC do not exclusively function in response expression but may also support appraisal processes [9]. Moreover, other studies also provide a foundation for the claim that the DLPFC contributes to subjective feelings, emotion, and self-awareness [42], [43]. The James-Lange theory of emotion defines emotion as perceived central representations of bodily responses to emotive stimuli, with emotional feelings dependent on bodily responses that may be generated automatically by the autonomic nervous system [43]. Specifically, our results are in line with the findings that the dorsal-caudal regions of the ACC/PFC are involved in the appraisal and expression of negative emotions and that the ventral- rostral portions of the ACC/PFC have a regulatory role in the limbic regions that are involved in generating emotional responses [9]. However, we also observed decreased GMVs of the DLPFC in anxiety patients without comorbid MDD, which differs from the CDA patient results. The development of depression is a particularly frequent complication across the range of anxiety disorders, which warrants further studies that delineate the processes behind the increased depression risk among individuals with anxiety disorders. Specifically, the right DLPFC is known to be more active during emotion suppression [44], [45], and the decreased GMV in this region may increase the vulnerability for pathological anxiety [46], [47]. However, the contrasting results may also be due to the relatively small group of subjects and the presence of various comorbid psychiatric diseases. The above results regarding the changes in the structure of the DLPFC require further research.

In addition to our ACC and DLPFC findings, we also demonstrated reduced left IFG GMVs in anxiety, CDA, drug-naïve, and adult patients. The IFG plays a critical role in anxiety and emotion regulation because it is connected to the amygdala, which is a key structure in the processing of anxiety in the brain [48]. Previous studies showed that the left IFG is involved in the adaptive response to social anxiety- provoking situations and has a relationship with SAD symptom severity [49]. The left IFG has also been implicated in the suppression of habitual behavior, and increased activity in this brain region was related to successful suppression of this behavior [50]. Therefore, decreased GMV in the left IFG in anxiety patients could be interpreted to be associated with a lack of sufficient inhibition in the emotional stimulus signaling of a potential threat that leads to mood disorders.

Medications, particularly those that influence the serotonin system, are hypothesized to desensitize the fear network at the level of the amygdala through its projections to the hypothalamus and brainstem, and they may also reduce cognitive misattributions at the level of the prefrontal cortex and hippocampus [31], [51]. Taking medication to be a potential confounding factor, we showed that the frontal cortex, insula, thalamus, and anterior cingulate in our drug-naïve subgroup constituted the fear circuitry model of PD [51]. The insula is connected to the thalamus and has projections to the prefrontal cortex, anterior cingulate gyrus, and hypothalamus; these connections also suggest that the insula plays a role in the processing of anxiety- related information in the brain [48]. Furthermore, the insula and thalamus are considered to be constituents of the visceral sensory cortex that detect increased heart rate and visceral arousal, which are often seen as characteristics of anticipatory anxiety [48], [51], [52]. Our results suggest that the two regions that belong to the arousal system may cause anxiety patients to be more easily aroused by emotional stimuli and display physiological anxiety.

Anxiety is a basic emotion that is present in infancy and childhood, and anxious children expect to experience a larger number of negative outcomes and events [53]. Neuroimaging studies have provided evidence that progressive structural brain abnormalities in children differ from those in adults [54], [55]. Our research excluded children and adolescents and did not demonstrate a significant effect of age on the results.

In accordance with previous definitions [35], the subtypes of anxiety show overlapping symptoms and share features of their threat-relevant responses (i.e., anxious apprehension, fear, and avoidance), but they differ with respect to the object and breadth of the recognized threat. SAD involves fear/avoidance, specifically that of social situations, but GAD and PD involve intrusive worries about diverse circumstances. Specifically, patients with GAD have clinical symptoms such as the fear of being outside or in open spaces from which escape would be difficult. Apart from these similar symptoms, other etiologies are shared by anxiety disorders, such as a low baseline high-frequency heart rate variability [56], decreased severity of anxiety symptoms during anxiety episodes over time [57], and less satisfaction with quality of life compared with non-anxious adults. Additionally, CDA but not anxiety was found to negatively impact the quality of life in these individuals [58].

## Limitations

There are some limitations in this study. First, our research is limited by the varied demographic and clinical characteristics of the samples and by the inclusion of few studies on the structural abnormalities associated with specific anxiety disorders, resulting in an insufficient statistical power and problems controlling for the anxiety in each of these groups. Second, the overlapped GMVs changes described in this paper are typical of the four types of anxiety disorders, but other comorbid psychiatric diseases such as OCD and separation anxiety may limit the power and reliability of GMV deficits findings in these areas. Given that the two studies included comorbid OCD patients account for small number of all the patients, the findings remained largely unchanged after discarding the two studies because they also comorbid depression. What's more, regional gray matter volumes in the dMFG/ACG were shared in patients with OCD and other anxiety disorders [5], so we supposed that including the comorbid OCD studies didn't affect the result significantly. In this study, we did not observe changes in GMV in the amygdala, which is associated with mood and anxiety disorders in humans [59], [60]. We speculated that this result is due to the relatively small sample size, although amygdala abnormalities are specific not only to different psychopathologies (e.g., borderline personality disorder) but also to their specific genotypes [61]. Thus, additional analyses (e.g., pattern classification) are needed to establish neuroanatomical markers as reliable diagnostic tools for anxiety disorders.

## Conclusions

Our meta-analysis results showed significant decreased regional GMVs in the right ventral anterior cingulate and the left inferior frontal gyrus in anxiety and CDA patients compared with controls by controlling for potential confounders such as antidepressants and age, and these decreased GMVs may represent a shared psychological dysfunction in anxiety disorders and CDA. The findings also suggest the importance of the ACC and PFC in emotion regulation, affectional expression, and physiological reactions, which are factors that contribute to the onset of anxiety disorders. Additional meta-analyses of a larger number of studies with high-quality evidence would aid us in identifying the most powerful predictors of anxiety disorders and in understanding their complex biological and psychological mechanisms and interactions that are involved in the onset of anxiety disorders.

## Supporting Information

Checklist S1 (DOC)
