# Supplementary material for: The Common Traits of the ACC and PFC in Anxiety Disorders in the DSM-5: Meta-Analysis of Voxel-Based Morphometry Studies
Source: PLoS One. 2014 Mar 27;9(3):e93432. doi: 10.1371/journal.pone.0093432 (PMC3968149; doi:10.1371/journal.pone.0093432)
Supplement: Checklist S1 — (DOC) [file pone.0093432.s001.doc]

| **Section/topic** | **#** | **Checklist item** | **Reported on page #** |
| --- | --- | --- | --- |
| **TITLE** | | |  |
| Title | 1 | The Common Traits of the ACC and PFC in Anxiety Disorders in the DSM-5: Meta-analysis of Voxel-Based Morphometry Studies |  |
| **ABSTRACT** | | |  |
| Structured summary | 2 | **Background**: The core domains of social anxiety disorder (SAD), generalized anxiety disorder (GAD), panic disorder (PD) with and without agoraphobia (GA), and specific phobia (SP) are cognitive and physical symptoms that are related to the experience of fear and anxiety. It remains unclear whether these highly comorbid conditions that constitute the anxiety disorder subgroups of the Diagnostic and Statistical Manual for Mental Disorders – Fifth Edition (DSM-5) represent distinct disorders or alternative presentations of a single underlying pathology. **Methods:** A systematic search of voxel-based morphometry (VBM) studies of SAD, GAD, PD, GA, and SP was performed with an effect-size signed differential mapping (ES-SDM) meta-analysis to estimate the clusters of significant gray matter differences between patients and controls. **Results:** Twenty-four studies were eligible for inclusion in the meta-analysis and constituted a total of 617 anxiety patients and 647 healthy controls. Reductions in the right anterior cingulate gyrus and the left inferior frontal gyrus gray matter volumes (GMVs) were noted in patients with anxiety disorders when potential confounders, such as comorbid major depressive disorder (MDD), age, and antidepressant use were controlled for. We also demonstrated increased GMVs in the right dorsolateral prefrontal cortex (DLPFC, including Brodmann areas 6/8/9/46) in comorbid depression-anxiety (CDA), drug-naïve and adult patients. Furthermore, we identified a reduced left middle temporal gyrus and right precentral gyrus in anxiety patients without comorbid MDD. **Limitation:** The difficulty in isolating the modulating effects of other anxiety disorders biased our research. **Conclusion:** Our findings indicate that a reduced volume of the right ventral anterior cingulate gyrus and left inferior frontal gyrus is common in anxiety disorders and is independent of comorbid depression, medication use, and age. This generic effect supports the notion that the four types of anxiety disorders have a clear degree of overlap that may reflect shared etiological mechanisms. The results are consistent with neuroanatomical DLPFC models of physiological responses, such as worry and fear, and the importance of the ventral anterior cingulate (ACC)/medial prefrontal cortex (mPFC) in mediating anxiety symptoms. | Abstract |
| **INTRODUCTION** | | |  |
| Rationale | 3 | Social anxiety disorder (SAD), generalized anxiety disorder (GAD), panic disorder (PD), agoraphobia (AG), and specific disorder(SP) are major anxiety disorders identified by the Anxiety, OC Spectrum, Posttraumatic, and Dissociative Disorders working group. | Introduction |
| Objectives | 4 | The principal aim of this study was to perform a search of all the published VBM studies on SAD, GAD, PD, AG, and SP to better understand whether these frequently co-occurring anxiety disorders may have, at least in part, a common etiology by controlling for the effects of antidepressants and age as potential confounders. The second aim of this study was to investigate the shared and unique neuroanatomical profile of anxiety and comorbid depression by discarding the patients who currently exhibit depression. | Introduction |
| **METHODS** | | |  |
| Protocol and registration | 5 | There is no review protocol similar to ours exists. | Methods |
| Eligibility criteria | 6 | Specify study characteristics (e.g., PICOS, length of follow-up) and report characteristics (e.g., years considered, language, publication status) used as criteria for eligibility, giving rationale. | Methods |
| Information sources | 7 | Potentially eligible studies that examined individuals with SAD, GAD, PD, AG, and SP were identified by conducting a search of the PubMed, ScienceDirect, and EBSCO databases between January 2001 (the date of the first VBM study in any anxiety disorder) and November 2013 | Methods |
| Search | 8 | Key words: “social anxiety” or “social phobia” or “SAD”, “generalized anxiety disorder” or “GAD”, “panic disorder” or “PD”, “specific phobia” or “SP”, “agoraphobia”, “phobia”, “stress disorder”, “anxiety”, and “Voxel-Based Morphometry” or “VBM” or “voxelwise” | Methods |
| Study selection | 9 | Two independent reviewers firstly assessed the titles and retrieved articles for relevance. Secondly, the articles remained eligible were assessed based on the abstract to determine whether any inclusion criteria were not met. The full text of all remaining articles were then assessed with a data extraction template, constructed for the purpose of organizing and extracting information from included articles. | Methods |
| Data collection process | 10 | First, we ensured that the same threshold was used throughout the entire brain in each included study to avoid biases toward liberally thresholded brain regions. Second, an effect-size signed map of the differences in gray matter was separately recreated for each study. Third, the mean map was obtained by calculating the mean of the study maps, weighting the means by the inverse of each study variance and accounting for inter-study heterogeneity. The statistical significance was assessed by performing a permutation test. | Methods |
| Data items | 11 | (a) author names, (b) date of publication, (c)categories of diseases and brain atlas, (d) subject group numbers, (e) comorbidities ,(f) medications used (including past and current antidepressants), (g) mean age with standard deviation, (h) ratio of gender, (i) coordinates associated with greater or smaller gray matter volume(GMV) in patients of anxiety compared with HC. | Methods |
| Risk of bias in individual studies | 12 | patients comorbid depression, use of antidepressants, age difference. | Methods |
| Summary measures | 13 | gray matter volume(GMV), the SDM value and number of voxels in the case vs. control comparison performed in this meta-analysis are reported. | Methods |
| Synthesis of results | 14 | effect-size signed differential mapping (ES-SDM) | Methods |

Page 1 of 2

| **Section/topic** | **#** | **Checklist item** | **Reported on page #** |
| --- | --- | --- | --- |
| Risk of bias across studies | 15 | comorbid depression, antidepressants and age. | Methods |
| Additional analyses | 16 | Discarded the patients comorbid depression to identify their difference, and also tested the putative modulating effect of antidepressants and age. | Methods |
| **RESULTS** | | |  |
| Study selection | 17 | 24 | Results |
| Study characteristics | 18 | Twenty-four studies were included in this meta-analysis according to the search criteria mentioned above and consisted of 5 studies on SAD; 3 studies on GAD; 13 studies on PD with and without GA; 2 studies on MDD and PD; 1 study on SAD, GAD, and separation anxiety disorder; and 1 study on SAD, GAD, and PD with and without MDD. | Results |
| Risk of bias within studies | 19 | 5 studies included patients with comorbid depression, 9 studies included participants who were taking antidepressant medication, 3 studies including childhood and adolescent subjects. | Results |
| Results of individual studies | 20 | These studies yielded a total combined sample of 617 subjects with comorbid depression-anxiety (CDA) and 647 HCs. | Results |
| Synthesis of results | 21 | anxiety patients showed decreased volumes in the right ventral anterior cingulate and left inferior frontal gyrus and increased volumes in the right precentral gyrus, right middle frontal gyrus, and right inferior parietal lobule compared with those of the HCs | Results |
| Risk of bias across studies | 22 | - | Results |
| Additional analysis | 23 | Anxiety Patients without Current Depression not only showed decreased regional GMVs in the right ventral anterior cingulate and left inferior frontal gyrus, which are areas that are also associated with CDA, but also revealed decreased GMVs in the left middle temporal gyrus and right precentral gyrus. The results of drug-naïve anxiety patients remained largely unchanged, with decreased GMVs found in the right ventral anterior cingulate and left inferior frontal gyrus, which are regions that were also found to exhibit decreased GMVs in CDA. Furthermore, the results also revealed a decreased GMV in the right insula and increased GMVs in the right precentral gyrus, right thalamus, right medial frontal gyrus, and left dorsal cingulate gyrus compared with healthy subject. Adult Patients with Anxiety Disorders exhibited decreased GMVs in the right ventral anterior cingulate and left inferior frontal gyrus and increased GMVs in the right precentral gyrus, right medial frontal gyrus, and right inferior parietal lobule. | Results |
| **DISCUSSION** | | |  |
| Summary of evidence | 24 | We present the first ES-SDM meta-analysis of VBM studies that examined the brain structural similarities of the anxiety subgroups of the DSM-5. After controlling for the effects of comorbid depression, antidepressants, and age, these anxiety disorders were found to share the same brain regions of decreased GMVs (namely, the right ventral ACC and left IFG) compared with controls. Therefore, the current pattern of results indicates that SAD, GAD, PD, and GA are not completely different entities at the level of their neuroanatomical phenotypes. This observation is simplistic, but lends further support to the argument that the anxiety subgroups share biological dimensions. | Discussion |
| Limitations | 25 | There are some limitations in this study. First, our research is limited by the varied demographic and clinical characteristics of the samples and by the inclusion of few studies on the structural abnormalities associated with specific anxiety disorders, resulting in an insufficient statistical power and problems controlling for the anxiety in each of these groups. Second, the overlapped GMVs changes described in this paper are typical of the four types of anxiety disorders, but other comorbid psychiatric diseases such as OCD and separation anxiety may limit the power and reliability of GMV deficits findings in these areas. Given that the two studies included comorbid OCD patients account for small number of all the patients, the findings remained largely unchanged after discarding the two studies because they also comorbid depression. What’s more, regional gray matter volumes in the dMFG/ACG were shared in patients with OCD and other anxiety disorders[5], so we supposed that including the comorbid OCD studies didn’t affect the result significantly. In this study, we did not observe changes in GMV in the amygdala, which is associated with mood and anxiety disorders in humans[59,60]. We speculated that this result is due to the relatively small sample size, although amygdala abnormalities are specific not only to different psychopathologies (e.g., borderline personality disorder) but also to their specific genotypes[61]. Thus, additional analyses (e.g., pattern classification) are needed to establish neuroanatomical markers as reliable diagnostic tools for anxiety disorders. | Discussion |
| Conclusions | 26 | Our meta-analysis results showed significant decreased regional GMVs in the right ventral anterior cingulate and the left inferior frontal gyrus in anxiety and CDA patients compared with controls by controlling for potential confounders such as antidepressants and age, and these decreased GMVs may represent a shared psychological dysfunction in anxiety disorders and CDA. The findings also suggest the importance of the ACC and PFC in emotion regulation, affectional expression, and physiological reactions, which are factors that contribute to the onset of anxiety disorders. Additional meta-analyses of a larger number of studies with high-quality evidence would aid us in identifying the most powerful predictors of anxiety disorders and in understanding their complex biological and psychological mechanisms and interactions that are involved in the onset of anxiety disorders. | Discussion |
| **FUNDING** | | |  |
| Funding | 27 | No current funding sources for this study |  |

*From:*  Moher D, Liberati A, Tetzlaff J, Altman DG, The PRISMA Group (2009). Preferred Reporting Items for Systematic Reviews and Meta-Analyses: The PRISMA Statement. PLoS Med 6(6): e1000097. doi:10.1371/journal.pmed1000097

For more information, visit: **www.prisma-statement.org**.

Page 2 of 2
